# Mental Health Difficulties and Help-Seeking Beliefs within a Sample of Female Partners of UK Veterans Diagnosed with Post-Traumatic Stress Disorder

**DOI:** 10.3390/jcm5080068

**Published:** 2016-08-01

**Authors:** Dominic Murphy, Emily Palmer, Walter Busuttil

**Affiliations:** 1 Combat Stress, Tyrwhitt House, Leatherhead KT22 0BX, UK; walter.busuttil@combatstress.org.uk; 2 King’s Centre for Military Health Research, King’s College London, London SE5 9RJ, UK; emily.palmer@combatstress.org.uk

**Keywords:** veterans, ex-service personnel, partners, spouses, carers, mental health, PTSD

## Abstract

In the UK there is a paucity of research about the needs of partners who are supporting ex-service personnel with mental health difficulties. In this study, we surveyed the mental health needs and barriers to help-seeking within a sample of partners of UK veterans who had been diagnosed with PTSD. Our sample included 100 participants. Forty-five percent met criteria for alcohol problems, 39% for depression, 37% for generalised anxiety disorder and 17% for symptoms of probable PTSD. Participants who met case criteria for depression, anxiety and problems with alcohol were more likely to report a greater number of help-seeking barriers. Participants who were experiencing mental health difficulties were more likely to endorse barriers connected to stigmatising beliefs than those associated with practical issues around accessing mental health services. The evidence presented suggests there may be a considerable burden of mental illness within this population. It would seem prudent to conduct further work to understand how best to address this clinical need.

## 1. Introduction

Since the start of conflicts in Afghanistan and Iraq in 2001 and 2002 respectively, there have been a number of ongoing research projects investigating the mental health of veterans who served on these deployments [1,2,3,4]. However, there has been less focus on the mental health of the intimate partners of UK veterans, and in particular, the partners of veterans who are suffering from mental health difficulties. A recent study of UK veterans seeking help for symptoms of post-traumatic stress disorder (PTSD) reported that between 53%–59% were in a relationship [5]. This suggests that there may be notable numbers of partners supporting veterans with PTSD in the UK. 

Studies in the US have shown that the romantic partners of those who deployed to the conflicts in Iraq and Afghanistan are at increased risk of experiencing mental health difficulties themselves [6,7]. A longer term effect has been shown between deployment and mental health difficulties in military partners. A study of Australian Vietnam veterans who had served 30 years previously, observed that for 11 out of 17 psychiatric diagnoses partners were at rates above general population levels [8]. Worryingly, within this ageing Vietnam veteran population, military partners were found to be at increased risk of suicidal ideation, planning and attempts than matched members of the general public [9]. In the above studies, a veteran having a diagnosis of PTSD has been demonstrated to be a significant predictor of psychological distress for their partner. Further, the severity of the veteran’s PTSD symptoms has also been shown to be a significant predictor to the extent of their partner’s compromised wellbeing [10,11,12,13,14]. This been observed to be the case even when controlling for socio-demographic differences, interpersonal violence and other health complaints [15]. 

Different explanations of the heightened risk in partners of veterans have been proposed. It has been suggested that partners of veterans with PTSD have a dual role in the relationship. Firstly, striving to preserve their relationship, and secondly, having to accommodate the illness into their lives, both of which have been argued to lead to feelings of isolation and emotional pressure [16]. Compelling evidence suggests that one of the factors contributing to poorer mental health in partners of veterans with PTSD is associated with feeling responsible for controlling daily stressors that could exacerbate the veterans’ PTSD symptoms [17,18,19]. Studies that explored the individual PTSD symptom clusters suggested that those associated with avoidance (e.g., emotional numbing) or arousal symptoms (e.g., sleep difficulties, irritability) were significant predictors of relationship difficulties and marital distress [13]. A further factor discussed within the literature is the impact of a veteran’s PTSD symptoms on their social functioning, and subsequent impact on the quality of their romantic relationships [20]. 

It has been reported that the partners of veterans with PTSD are themselves at increased risk of experiencing symptoms that mirror those of PTSD [21]. Sometimes labelled ‘secondary traumatic stress’, this refers to trauma reactions similar to PTSD linked to listening to reports of a family member’s exposure to suffering [22]. It has been observed that partners’ symptoms of secondary traumatic stress appear to be associated with having fewer members in their social network [22].

Research in US samples has demonstrated that partners of veterans with PTSD are more likely to score higher levels of psychological distress than partners of individuals with other mental health difficulties [23]. Direct comparisons between studies of veteran and non-veteran intimate partners is difficult due to differing metrics being employed. However, two studies that used the same measure reported that 28% of caregivers of relatives with dementia met case criteria for common mental health difficulties, compared to 45% of caregivers of military relatives with PTSD [12,24]. A meta-analysis that explored PTSD, veteran status and relationship quality observed a stronger effect size between PTSD and partners’ psychological distress in veteran compared to non-veteran groups [25].

Little is known about the help-seeking behaviour of military partners who experience mental illness. In the UK veteran population only around 25% of those with probable PTSD report seeking help [26,27]. These rates are not dissimilar to the rates of those who screen positive for PTSD seeking helping in the UK general public, European samples and US samples [28,29,30]. The most common barriers that have been suggested that may act to prevent help-seeking fall with two main categories: those related to stigmatising beliefs and those associated with practical access issues [31]. Examples of stigmatising beliefs are feeling embarrassed about seeking help or being seen as weak by others. Examples of practical access issues are not being able to get time off work to attend appointments or not knowing how to seek help.

Evidence has suggested that mental health issues are viewed negatively within the Armed Forces [32]. Those experiencing physical illness are viewed as more legitimate than those experiencing a mental illness [33]. It is proposed that these attitudes are internalised to form negative beliefs about mental illness [34] and therefore about the self in those experiencing mental health problems [35]. This in turn has a negative impact on self-esteem [36]. Such beliefs are reported to be the biggest barrier to accessing mental health support [37]. Some studies suggest that stigmatising beliefs extend to other family members. One study found an association between children accessing mental health support and concerns by their parents that this may impact their military careers [38].

Whilst, stigma is reported to be the biggest barrier, practical access issues should not be under-estimated [34]. Practical access barriers have also been endorsed by samples of serving and ex-military personnel [27]. Further, it was found that these barriers did not reduce after leaving the military. It is not known whether military or veteran partners experience such barriers. It is possible that in the population of partners of veterans with PTSD, increased caring responsibilities may lead to greater practical barriers. This study aimed to explore the assumption that military partners may also struggle to engage in appropriate mental health services due to stigma-related and practical barriers.

The above evidence suggests that the partners of veterans with PTSD are at increased risk of experiencing psychological distress. It should be noted that the majority of the research reviewed above is restricted to US or Australian samples. Work by the Mental Health Foundation demonstrated the paucity of research in the UK to understand the mental health needs of partners of veterans who have mental health difficulties [39]. In the current study we explored the mental health needs of a sample of partners of veterans who were accessing a mental health service for symptoms of PTSD. Our primary aim was to explore prevalence of depression, anxiety, trauma and alcohol related mental health issues in intimate partners of veterans. Our secondary aim was to explore barriers to accessing support for mental health in this group.

## 2. Materials and Method

### 2.1. Setting

We drew the sample for this study from UK veterans who were accessing support for mental health difficulties from an organisation called Combat Stress. Combat Stress is a national mental health charity in the UK that provides clinical services to veterans. Outside of the UK’s National Health Service (NHS) it is the largest provider of mental health services to veterans and has been funded by the NHS to provide a national specialist service for veterans with PTSD. Combat Stress was selected as a place to recruit because it is a national service that specialises in treating UK veterans who have been diagnosed with PTSD.

### 2.2. Participants

Data collection for this project was complicated by the fact that we had to ask consent from veterans to contact their partners. Between the start of 2013 and mid 2014, 333 veterans had been treated by Combat Stress for PTSD and been successfully followed up six months after the end of treatment. From this sample, 197/333 (59%) reported having a partner. We attempted to contact this group of 197 veterans to request consent to contact their partners. Contact was made via mailing out opt-in letters to veterans that explained the rationale for the study, made it clear that all information collected would be confidential and not shared with either partner, and asked for consent and the contact details of their partner. We were able to get consent from 141/197 (72%) of this group of veterans. This group of veterans had a mean age of 45.1 years. Eight per cent had been in the Royal Navy, 84% in the Army and eight per cent in the Royal Air Force. Seven per cent had left the Armed Forces with an Officer rank and 93% were at other ranks. There were 41% in employment and 82% had low educational attainment of GSCEs or below. Such demographics are typical of this population based on previously published studies [5]. This group of 141 partners formed the sample for the current study. Data were collected via sending questionnaires to potential participants. Questionnaires were sent out via two waves of mail outs to encourage as many partners to participant as possible.

### 2.3. Measures

The questionnaire asked participants to provide answers to a number of demographic questions. These included, age, sex, length of relationship with veteran partner, whether they were living with their veteran partner, whether the participant was either a current or ex-member of the Armed Forces themselves, whether they were in employment, if they had dependents and if they had any physical health problems. Participants were also asked whether they had previously sought support for mental health difficulties and to endorse or not a list of eight potential barriers to seeking help. These potential barriers broadly fell into two categories, practical issues (for example concerns a ‘difficultly getting time off work’) and stigmatising beliefs (for example concerns about ‘worrying about what others would think of me’) about seeking help for mental health difficulties. These potential barriers were selected based on previous research and clinical experience with veteran partners. Four of the barriers were selected and used verbatim from a previous study exploring help seeking in veterans [27]. The remaining four were created to test assumptions about the limitations posed by probable caring responsibilities, for dependents and their veteran partner, and to investigate specifically barrier relating to isolation and fear. 

In addition, participants were asked to complete a range of mental health outcome measures. These included the nine item Patient Health Questionnaire (PHQ-9) which measures symptoms of depression and can be used to assess severity. A score of 10 or above has been noted as indicative of meeting case criteria for depression [40,41]. The seven item Generalised Anxiety Disorder Assessment (GAD-7) was used to explore symptoms of generalised anxiety and score of eight or above can be used to define meeting case criteria [42]. Symptoms of PTSD were measured using the 10 item Trauma Screening Questionnaire (TSQ). The TSQ assesses whether a participant has been exposed to a traumatic event in the past and then for those participants who have, they are asked to complete a range of questions assessing for probable PTSD. A score of six or more has been used to define probable PTSD [43]. The three item AUDIT-C was used as a screening tool for problems with alcohol, a score of three or above for females or four and above for males is used to define the presence of alcohol problems or not [44,45].

### 2.4. Analysis

The first stage of the analysis was to describe the demographic characteristics of the sample. The mental health profile of the sample was then described with mean total scores and the proportion of participants whose scores met case criteria presented. Following this, regression analysis was conducted to explore the whether the demographic differences impacted on the measures of mental health. Multivariate regression models were fitted and beta co-efficient and 95% confidence intervals calculated to explore whether there was a relationship between each demographic characteristic and the mental health scores. The multivariate regression models included all the demographic variables in the table.

Proportions of the sample endorsing the eight different barriers to seeking support were calculated. Two of the barriers were screened for non-applicability. The first of these was related to getting time off work and participants not in employment were removed from the analysis. The second was related to childcare issues and participants without children living at home were removed from the analysis. Mann-Whitney U tests were used to explore whether differences were present between the total number of barriers reported between participants who met case criteria for each health outcome and those who did not. The final stage of the analysis assessed the associations between endorsing a particular barrier to care and severity of mental health scores. These analyses were restricted to the mental health outcome that had the highest level of significance in the previous set of analyses that explored the differences between the total number of barriers endorsed by mental health outcome. Univariate logistic regression models were fitted and odds ratios and 95% confidence intervals calculated between each barrier and severity of mental health scores. Two different multivariable regression models were then fitted which built upon each other in a stepwise fashion. The first adjusted for demographics related to the participant (age, employment status, whether they had children living with them and whether they reported having a physical health problem); the second further added demographics related to their relationship (whether they were living with their veteran partner or not and the length of their relationship). All analyses were conducted using STATA 13 (StataCorp, College Station, TX, USA).

### 2.5. Ethical Approval

All subjects gave their informed consent for inclusion before they participated in the study. The study was conducted in accordance with the Declaration of Helsinki, and the protocol was approved by the Combat Stress Ethics Committee.

## 3. Results

Questionnaires were sent to our sample of 141 partners of veterans who had accessed Combat Stress for support with symptoms of PTSD. Of these we received 100 responses which gave us a response rate of 70.9%. All 100 participants were female and in relationships with male veterans. The mean age was 46.0 years old, 62% of the sample was working, 54% had children living with them, 5% had previously been in the military themselves and 41% reported experiencing physical health problems. In terms of their relationships with the veterans, 88% reporting living with the veteran and the mean length of relationship was 16.0 years.

The prevalence rates of a range of health outcomes were reported in Table 1. Forty-five percent of the participants scored above the cut-offs for alcohol difficulties, 39% for symptoms of depression, 37% for generalised anxiety and 17% for probable PTSD. In general, socio-demographic characteristics did not seem to predict severity of mental health presentations. However, not working was found to increase the severity of symptoms of depression, anxiety and PTSD. Living with a veteran was associated with experiencing higher levels of anxiety. Lastly, having previously served in the military was observed to be protective for symptoms of depression and anxiety (Table 2).

Participants were asked to endorse or not a list of eight possible barriers to seeking help for mental health difficulties, these barriers were broadly categorised to fall within either practical access barriers or barriers related to stigmatising beliefs. Their responses are given in Table 3. Not feeling as if their difficulties were a priority and fearing that other people would not understand them were the most commonly endorsed barriers. This was followed by issues related to difficulty having time off work, childcare issues and being unsure where to seek help. There seemed to be a trend that barriers connected to practical access barriers were more frequently reported than those associated with stigmatising beliefs. The analyses in Table 4 explored whether the total number of barriers endorsed differed between participants who met case criteria on the range of health outcomes and those who did not. Participants who met case criteria for depression and generalised anxiety disorder were more likely to report more barriers to seeking support for mental health difficulties than those who did not. Whilst not significant, the p value approached significance for those screening positive for probable PTSD, also reporting a greater number of barriers. The final stage of analysis was to explore which individual barriers were more likely endorsed or not between participants scoring above or below the cut-off for generalised anxiety disorder (Table 5). The participants who suffered from generalised anxiety disorder were more likely to endorse three of the eight barriers. These barriers were associated with stigmatising beliefs (‘I am worried about what others would think of me’, ‘others would not understand my needs’ and ‘I am too embarrassed to seek help’).

## 4. Discussion

The results of this study suggest there may be high prevalence rates of mental health difficulties within a sample of partners of UK veterans who had a diagnosis of PTSD. Forty-five percent of the sample screened positive for problems with alcohol, just under 40% of the sample met criteria for problems with depression or generalised anxiety and 17% screened positive for probable PTSD. When looking at symptoms of anxiety and depression, the rates observed within this study seem higher than the prevalence rate of 20% that has been reported for studies of the UK general public [46], and higher than rates of depression of 28% that have been reported in a sample of caregiving partners of suffers of dementia [24]. What accounts for this difference is unclear. Previous research suggests the veteran-specific context may account for the increased burden of mental health difficulties for caregiving partners of veterans versus other caregivers [17,18,21,22,47,48]. Authors have also noted that the caregivers of sufferers of PTSD could be at increased risk because PTSD is associated with interpersonal problems and impaired social functioning [13,20,47,49]. This possible explanation seems particularly pertinent within the current population as significant periods of time between leaving the military and seeking help (over 13 years) have been reported, as well as high levels of functional impairment [5]. So it may be a combination of caring for an ex-member of the Armed Forces, who is also suffering from PTSD, which accounts for the high burden of mental illness reported within this sample.

As discussed above, the results from this paper indicate a significant burden of mental illness with caregivers of veterans with PTSD. We also observed that many in our sample endorsed barriers to seeking support for mental health difficulties. Our results suggest that practical access barriers were more likely to be reported than barriers connected to stigma-related beliefs in the overall sample. However, evidence was presented that showed that participants who met case criteria for depression and generalised anxiety were likely to endorse a greater number of barriers to help-seeking than participants who did not. Further, they were more likely to endorse barriers related to stigmatising beliefs than those related to practical access issues. This finding replicates similar research within military populations that reported a similar relationship [26,50,51].

### 4.1. Clinical Implications for Caregiving Partners

A review of the services for veterans’ partners in the UK indicated that the support available to partners primarily consists of peer-support groups, and that these are only offered in a modest number of areas across the UK. Positively, a recent review article concluded that there is tentative evidence to indicate the positive impact of peer-led groups [52]. However, the authors of this report also discussed that there were methodological issues with many of the studies that they reviewed in this area [52]. The high prevalence of mental health difficulties presented within the current study implies the need for more structured and evidence-based clinician-led interventions. The findings indicated a range of mental health difficulties including anxiety, depression, trauma and alcohol use. This implies the need for support that is tailored to complex difficulties and co-morbidity, and that is flexible around an individual partner’s presentation. As discussed within the introduction, there is evidence within the literature that the caregivers of veterans appear to be at increased risk of difficulties compared to other caregiving groups [23]. As such, this may indicate that any support offered to them may need to address their veteran-specific context. Evidence from the US indicates that structured programmes, that include elements of psycho-education, symptom management, support around caring for veterans with mental health difficulties and support to engage with statutory mental health services have been successful for reducing caregiver distress for veteran partners [53,54,55]. To date, less research has been conducted with UK samples. There is evidence to suggest the influential role a partner could play in the success of a veteran’s treatment outcomes [56]. Given this, providing clinical support that is in a timely manner that co-ordinates with veteran treatment will be important. In addition, our findings suggest that any intervention aimed at supporting the caregiving partners of veterans will have to consider how to overcome practical issues that may present barriers to attendance. Given that those meeting criteria for common mental health difficulties were more likely to endorse barriers relating to stigma, any programme will need to be designed to overcome these barriers presented by these beliefs. This may involve outreach to improve accessibility for partners, as stigmatizing beliefs may reduce their ability to proactively seek support. Programmes may also need to incorporate exploring and challenges such beliefs in the early stages to maintain on-going engagement of partners.

### 4.2. Clinical Implications for Veterans

In the current study, our sample was drawn from the partners of veterans who had received a diagnosis of PTSD. The importance of increasing understanding about the mental health needs of caregiving partners is supported by research that indicates the influential role a partner caregiver may have in the success of a veterans’ treatment. For example, the importance of family environment in recovery from chronic PTSD has been demonstrated. Studies have shown an association between unstable environments and the maintenance of PTSD [57] and that living in an environment with high levels of hostility is associated with poorer treatment outcomes of PTSD [58]. Further, marital distress has been demonstrated to predict negative PTSD treatment outcomes [13] and veterans with PTSD who reported strong functioning of their intimate relationships have greater treatment success [56]. Beyond the environment a veteran is surrounded by, there is evidence that actively involving a significant other in PTSD treatment programmes improves outcomes [59]. A relationship between increasing a veteran partner’s knowledge of PTSD and a reduction in the severity of PTSD symptoms for the veteran has been observed [60]. These findings suggest that paying attention to a partner’s distress, whilst providing treatment for a veteran’s PTSD is important. Further research is needed to understand the most effective type of support; as indicated by above, supporting the partner to decrease stress in the home environment and psycho-education have been demonstrated as potential areas to focus on during interventions that aim to support the veteran’s treatment by treating their partner.

### 4.3. Strengths and Weaknesses

This is the first study of its kind in the UK to explore the mental health from a sample of caregiving partners of veterans seeking support for mental health difficulties. The study profited from drawing its participants from a sample of veterans that had all received a diagnosis of PTSD, as this increases the ecological validity of the findings to understand the needs of partners of those veterans with chronic and complex presentations. In addition, the study included veterans from a range of conflicts, which closely align to those most frequently deployed on by help-seeking UK veterans [61].

There are a number of limitations that need to be considered when interpreting the observed results. One hundred percent of the sample was female and reported being partners to male veterans. This may reflect the predominantly male veteran population but does mean that male caregiving partners of female veterans or partners that are not in heterosexual relationships were not represented in the study sample. Whilst these might only be minority groups, the very nature of being a minority group, coupled with the difficulties of being a caregiver for a veteran with mental health difficulties, may mean that these subgroups could have unique needs that need exploration. The average length of relationships in the current study was 16.0 years which could indicate a bias towards recruiting those partners in more stable long-term relationships, though it is worth noting at 23% of the sample reported being in a relationship that was less than four years long. One explanation could be because, as mentioned above, on average it takes veterans 11.8 years after they have left service before they seek support from Combat Stress which means that they are more likely to be older and hence had the opportunity to be in a relationship for longer [61]. An alternative explanation could be that veterans in more unstable relationships have separated by the time they seek support. As such, this could mean that caregiving partners in shorter term relationships and those partners where the relationship is breaking down may have been under-represented in the current study. Whilst not strictly a limitation, an important consideration is that the sample was recruited from a population of veterans that have a diagnosis of PTSD, where the majority also meet criteria for a range of other co-morbid mental health difficulties and that there is evidence of high degrees of functional impairment. As such, the findings within the current study about the high burden of mental illness within the partners of veterans who themselves have complex presentations, but may be not reflective of the mental health of the partners of veterans who are suffering from less severe mental health difficulties. Partners were recruited for the study from veterans who had engaged on a treatment programme. As such, this could have primed them to be more willing to seek help themselves and could present a limitation to the generalisability of the findings around barrier to help-seeking for the wider veteran partner community.

### 4.4. Conclusions

Despite these limitations, this study is the first of its kind to explore the mental health of caregiving partners of UK veterans with PTSD. The findings presented provide evidence of the high burden of mental health difficulties within this sample and demonstrate the need to develop services specifically for this client group that take into account the veteran-specific nature of their experiences. In this study, participants who themselves suffered from mental health problems reported a greater number of barriers. Further, the barriers they reported tended to be associated with stigma-related beliefs rather than practical access barriers. The sample used in this study was recruited from partners of veterans with a diagnosis of PTSD. It would be helpful if future studies explored the mental health of a sample of partners drawn from a non-clinical community sample to explore whether being the partner of a veteran increases their risk of mental illness over and above that of non-veteran partners. This may elucidate further the impact of the veteran-specific context.

## Figures and Tables

**Table 1:** Describing mental health outcomes scores and numbers meeting case criteria.

Health Outcomes	Case (%)	Mean Score (95% CI)
*Depression*		
PHQ-9 (10+)	39/100 (39%)	8.59 (7.21–9.97)
*Generalised Anxiety*		
GAD-7 (8+)	37/100 (37%)	7.19 (6.04–8.34)
*PTSD*		
TSQ (6+)	17/100 (17%)	3.82 (3.04–4.60)
*Alcohol problems*		
AUDIT-C (3+)	45/100 (45%)	2.72 (2.25–3.19)

**Table 2:** Associations between demographic characteristics and scores on a range of health outcomes.

Characteristic	PHQ-9	GAD-7	AUDIT-C	TSQ
	Adjusted β ^a^ (95% CI)	Adjusted β ^a^ (95% CI)	Adjusted β ^a^ (95% CI)	Adjusted β ^a^ (95% CI)
*Age*				
Per year	−0.02 (−0.21, 0.18)	−0.07 (−0.23, 0.10)	0.05 (−0.01, 0.12)	0.02 (−0.10, 0.13)
*Physical Health*				
Yes	2.69 (−0.33, 5.72)	1.15 (−1.39, 3.69)	−0.42 (−1.45, 0.62)	−0.17 (−1.96, 1.62)
*Employment status*				
Not working	4.16 (1.07, 7.25) *	3.51 (0.92, 6.10) *	−0.20 (−1.22, 0.82)	1.85 (0.01, 3.70) *
*Children or not?*				
Living at home	2.78 (−0.28, 5.84)	3.12 (0.55, 5.69)	1.03 (0.05, 2.03) *	0.67 (−1.05, 2.39)
*Ex-service history*				
Yes	6.31 (0.09, 12.5) *	4.23 (1.00, 9.45) *	0.94 (0.12, −1.20)	−0.94 (−3.12, 1.24)
*Relationship status*				
Living with partner	2.07 (−2.63, 6.77)	5.12 (1.18, 9.07) *	−0.38 (−1.93, 1.18)	−0.11 (−2.97, 2.75)
*Relationship length*				
Per year	−0.05 (−0.21, 0.11)	0.01 (−0.13, 0.13)	−0.06 (−2.01, 4.80)	0.01 (−3.57, 7.68)

^a^ Adjustment made for all other variables in table. * *p* ≤ 0.05.

**Table 3:** Rates of participants endorsing barriers to seeking help.

Help-Seeking Barrier	N (%)
*Practical Access issues*	
Difficultly getting time off work ^1^	19/61 (31%)
Childcare issues ^2^	16/54 (30%)
Unsure of where to get help	27/99 (27%)
I do not feel it is a priority	37/99 (37%)
*Stigmatising beliefs*	
I am scared to seek help	5/99 (5%)
Others would not understand my needs	34/99 (34%)
Worried about what others would think of me	20/99 (20%)
I am too embarrassed to seek help	14/100 (14%)

^1^ Restricted to participants in employment; ^2^ Restricted to participants with children living at home.

**Table 4:** Describing differences between total number of barriers endorsed between participants meeting case criteria or not on a range of health outcomes.

Health Outcome	Not Case, *n*	Case, *n*	*p* Value
*Depression*			
PHQ-9 (10+)	1.52	2.16	0.05 *
*Generalised Anxiety*			
GAD-7 (8+)	1.35	2.46	0.01 *
*PTSD*			
TSQ (6+)	1.62	2.47	0.07
*Alcohol problems*			
AUDIT-C (3+)	1.98	1.5	0.22

* *p* ≤ 0.05.

**Table 5:** Exploring associations between endorsing a specific barrier and meeting case criteria on the seven item Generalised Anxiety Disorder Assessment (GAD-7).

Help-Seeking Barrier	Unadjusted Model	Model 1	Model 2
	Odds Ratio (95% CI)	Odds Ratio (95% CI)	Odds Ratio (95% CI)
*Practical Access issues*			
Difficultly getting time off work ^1^	1.64 (0.52, 5.24)	1.54 (0.45, 5.37)	1.37 (0.37, 5.08)
Childcare issues ^2^	2.20 (0.67, 7.23)	2.15 (0.59, 7.86)	1.90 (0.49, 7.47)
Unsure of where to get help	1.86 (0.76, 4.57)	1.75 (0.68, 4.53)	1.74 (0.63, 4.75)
I do not feel it is a priority	1.79 (0.77, 4.12)	1.43 (0.58, 3.51)	1.87 (0.69, 5.11)
*Stigmatising beliefs*			
I am scared to seek help	7.39 (0.79, 68.9)	12.7 (0.57, 28.3)	11.4 (0.54, 27.4)
Others would not understand my needs	3.31 (1.39, 7.88) *	3.49 (1.37, 8.87) *	3.87 (1.44, 10.4) *
Worried about what others would think of me	1.93 (0.91, 5.19)	2.13 (0.93, 6.17)	2.85 (1.00, 9.17) *
I am too embarrassed to seek help	5.37 (1.54, 18.7) *	6.08 (1.59, 23.3) *	5.85 (1.45, 23.6) *

* *p* ≤ 0.05. ^1^ Restricted to participants in employment. ^2^ Restricted to participants with children living at home. Model 1 β adjusted for age, employment status, child or not and physical health. Model 2 the same as model 1 further adjusted for living with partner and length of relationship.
